# A Rare and Rapidly Progressive Case of Primary Esophageal Malignant Melanoma in an Elderly Patient

**DOI:** 10.7759/cureus.74056

**Published:** 2024-11-19

**Authors:** Patrícia Sobrosa, Maria Inês Risto, Rita Mota, Joana Couto, Luciana Sousa

**Affiliations:** 1 Internal Medicine, Unidade Local de Saúde do Alto Minho, Viana do Castelo, PRT

**Keywords:** dysphagia, esophagus melanoma, geriatric palliative care, noncutaneous melanoma, primary malignant melanoma

## Abstract

Primary malignant melanoma of the esophagus (PMME) is a rare malignancy typically associated with poor prognosis, particularly in elderly patients. Here, we present the case of an 85-year-old female patient with a three-month history of progressive dysphagia and heartburn-related epigastric pain. Endoscopy revealed a polypoid esophageal lesion, confirmed as melanoma via biopsy with positive immunohistochemical staining for Melan-A and SOX-10. Given her age, frailty, and comorbidities, she was deemed unfit for surgery. A palliative approach, focusing on symptom management and systemic therapy, was adopted. Unfortunately, her condition rapidly worsened, leading to severe malnutrition and emaciation. The treatment focus shifted exclusively to symptomatic relief and best supportive care, and she ultimately passed away six months after diagnosis.

PMME is rare and its diagnosis is challenging, especially in elderly patients; this case emphasizes the importance of individualized treatment plans. Early detection remains difficult due to the asymptomatic nature of early-stage disease. Treatment strategies are limited and should be carefully individualized, particularly in older patients, where the risks of aggressive intervention may outweigh potential benefits. In this group of patients, the emphasis should be placed on quality of life rather than curative intent.

## Introduction

Melanomas are primarily skin tumors, with mucosal melanomas being rare and representing 0.8%-3.7% of all melanomas [1]. Primary malignant melanoma of the esophagus (PMME) is an extremely rare subtype, accounting for less than 0.05% of all melanoma cases [2,3]. Primary mucosal melanoma can affect any epithilium; however, it is most commonly found in the vulvovagina, anorectum, oral cavity, nasal cavity, and sinuses [1,4]. Thus, PMME is a rare type of esophageal cancer, accounting for 0.1%-0.8% of all primary esophageal cancers [2]. Its rapid progression and poor prognosis are key features, particularly in elderly patients. PMME is most commonly reported in patients aged 60-70 years, with some studies indicating a 2:1 male predominance, though demographic variability may exist [5,6].

In 90% of cases, it reveals as solitary lesions involving the mid and distal portions of the esophagus, presenting with multiple lesions only in 13% cases [2,3,7]. Macroscopically, endoscopy reveals polypoid and vegetating lesions that ultimately cause luminal obstruction and esophageal stenosis due to intrinsic growth. In terms of color, 85% of cases show hyperpigmented lesions, with the remaining appearing amelanotic [2,3,5]. One of the challenges in diagnosis is distinguishing PMME from esophageal melanosis, which is a pre-cancerous condition [2].

As most of the signs and symptoms are nonspecific, diagnosis is often delayed. PMME is a complex diagnosis requiring the analysis of signs and symptoms, diagnostic exams, biopsy and histological/immunohistochemical studies [5,6]. At the time of diagnosis, 30%-40% of patients present with metastasis, more commonly in locoregional lymph nodes (40%-80%), such as the periesophageal, mediastinal and celiac lymph nodes [3,5]. Moreover, more than 90% of the tumors are larger than 2 cm at diagnosis [7]. When compared to cutaneous melanomas, this disease is associated with high morbidity and unfavorable prognosis [2,7]. It has an overall survival rate at five years of less than 5%, and a median 10-month survival rate with a disease-related mortality of 85% [2,3,7]. Given the rarity and morbidity of PMME, treatment standardization is challenging.

For over a century, there has been an ongoing debate on the origin and treatment of PMME. This report aims to examine the latest developments in therapy, especially immunotherapy, and explore future perspectives in managing this challenging disease [2]. We discuss the case of an 85-year-old woman diagnosed with PMME, whose advanced age, frailty, and comorbidities posed additional management challenges, highlighting the difficulties and therapeutical limitations in treating elderly patients with this aggressive malignancy.

## Case presentation

An 85-year-old woman, with a medical history of hypertension and dyslipidemia, was admitted to the emergency department with a three-month history of right hypochondrial pain radiating to the epigastrium, nausea and vomiting not related to food intake. Initially, she was treated with antispasmodics with partial improvement seen.

**Figure 1:**
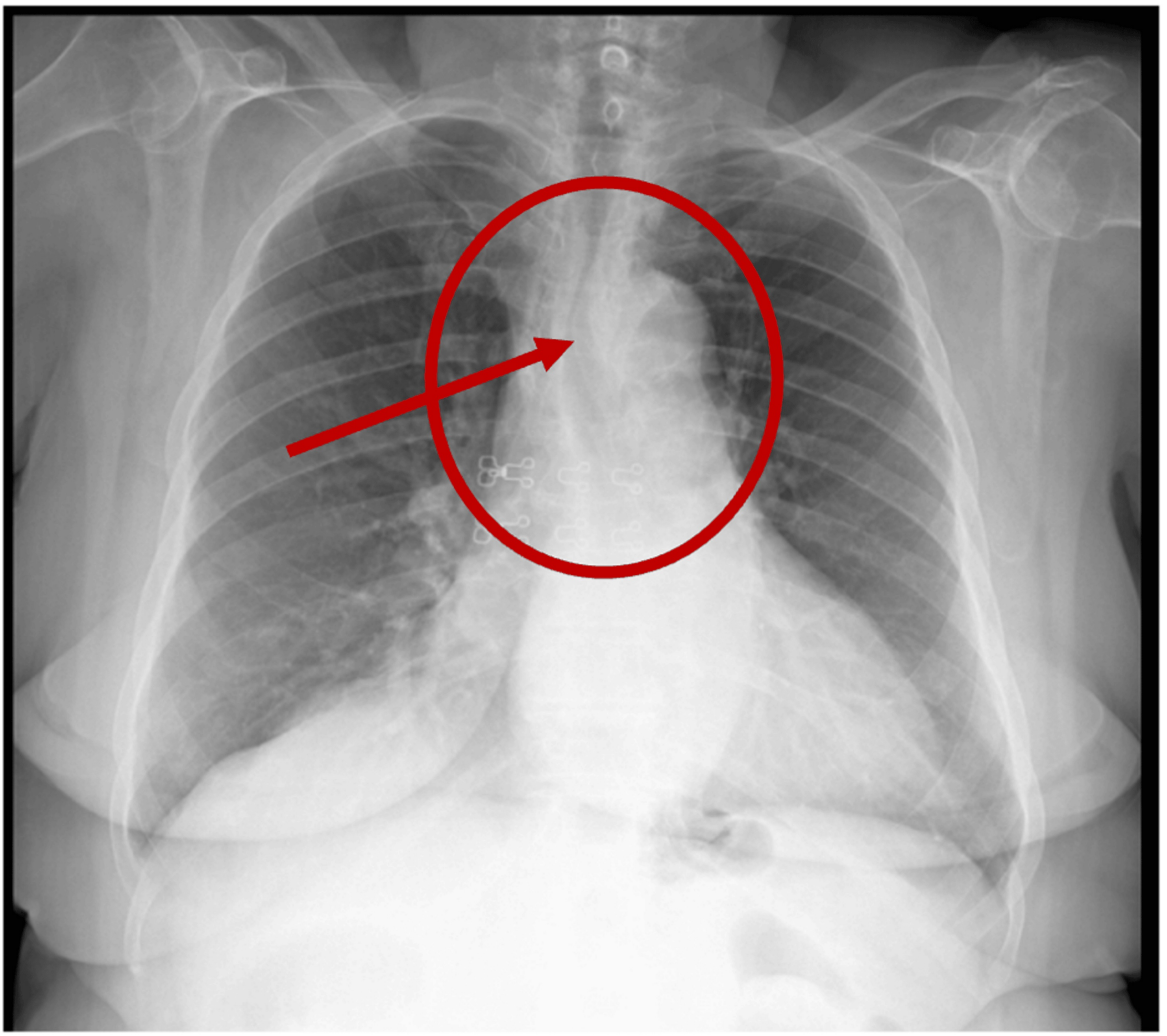
Chest X-ray showing mediastinal widening (red circle) and tracheal deviation (red arrow)

One month after the beginning of these symptoms, she developed rapidly progressing dysphagia and significant weight loss of 10 kg. Upon admission, laboratory tests revealed normocytic anemia (hemoglobin 11.7 g/dL), without vitamin deficiencies or other abnormalities. Chest X-ray revealed mediastinal widening and tracheal deviation (Figure 1).

**Figure 2:**
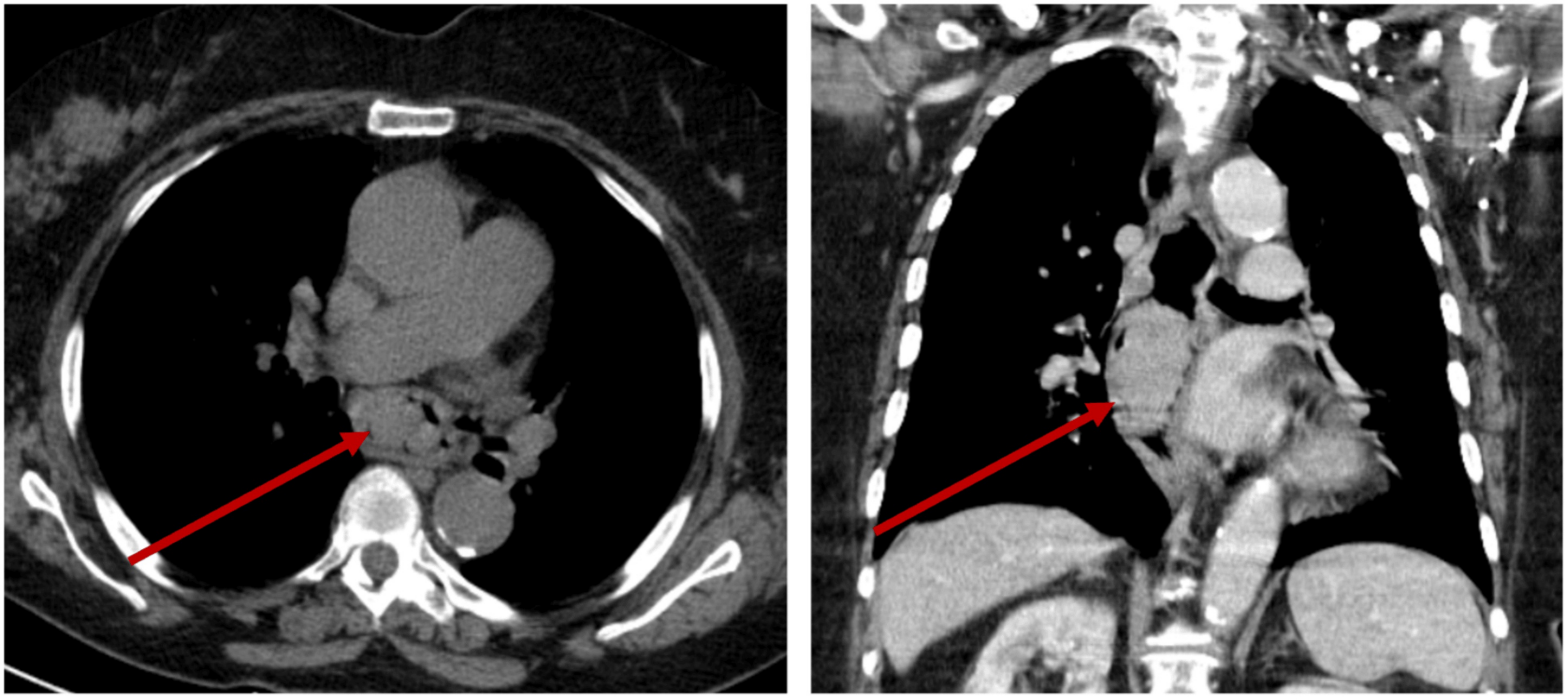
A CT scan showing the exophytic expansive lesion in the middle third of the esophagus, measuring approximately 3.7 x 3 cm axially, with a 5.3 cm craniocaudal dimension, with homogeneous contrast enhancement (red arrow)

A computed tomography (CT) scan showed an exophytic expansive lesion in the middle third of the esophagus, measuring approximately 3.7 x 3 cm axially with a 5.3 cm craniocaudal dimension, with homogeneous contrast enhancement. There was no evidence of extraluminal extension or adenopathy (Figure 2).

**Figure 3:**
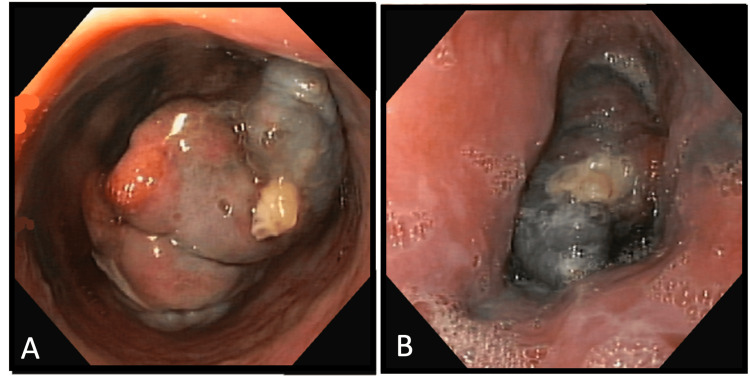
Upper endoscopy showed a 35-mm polypoid, with a base of insertion in varicose cords, at 28 cm from the incisors, extending to the esophagogastric junction (A). The lesion appeared violaceous/black, vascularized and friable (B).

For further clarification, an upper endoscopy was performed, revealing a large 35-mm polypoid lesion with a base of insertion in varicose cords, located 28 cm from the incisors, extending to the esophagogastric junction. The lesion appeared violaceous/black, vascularized and friable. It occupied two-thirds of the luminal circumference, without causing total obstruction, allowing the endoscope to pass through (Figure 3).

**Figure 4:**
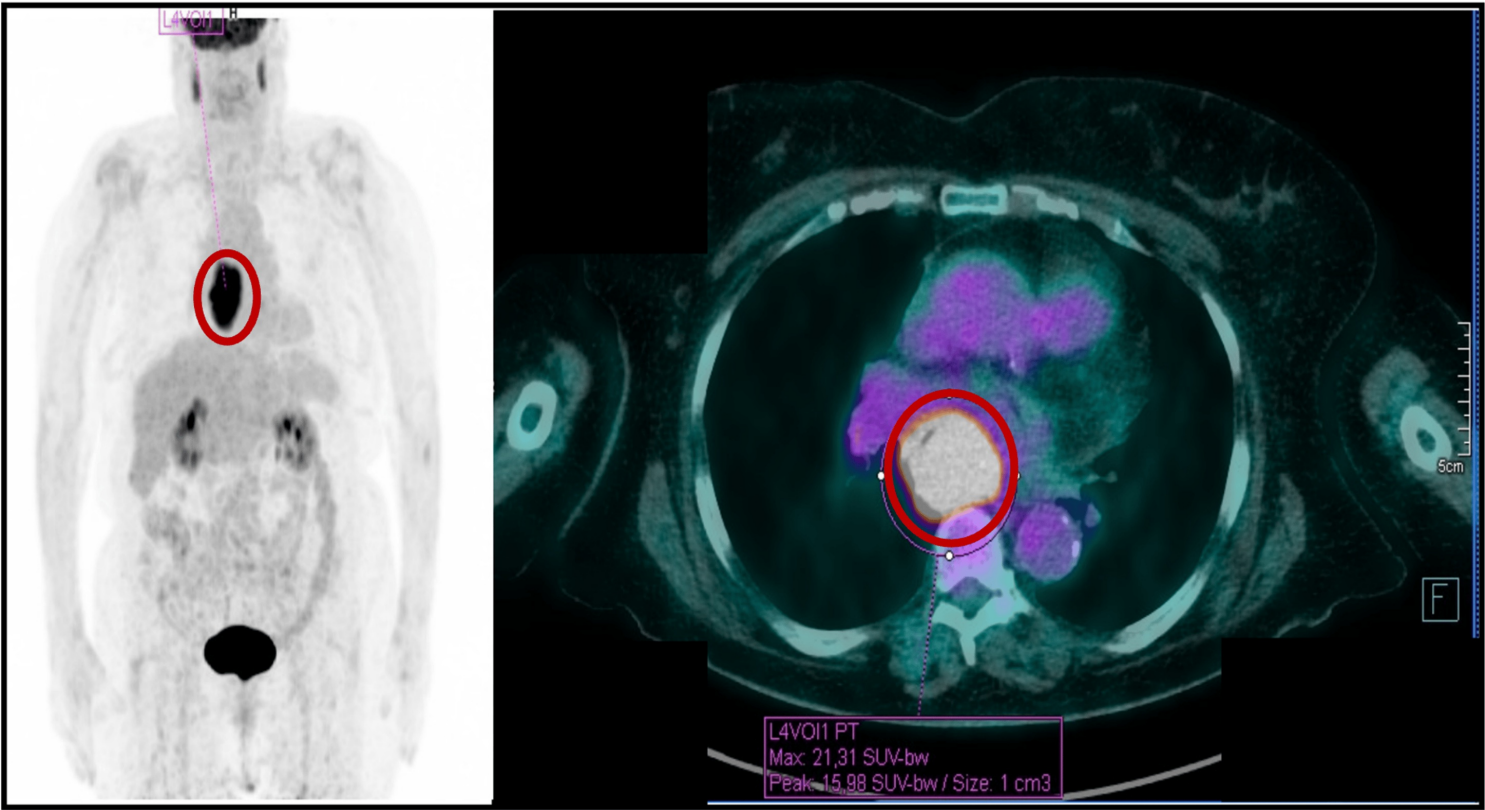
A PET-CT scan showing intense uptake in the lesion located in the middle/distal third of the esophagus (red circle), with an SUVmax of 21.3 PET-CT, positron emission tomography/computed tomography; SUVmax, maximum standardized uptake value

Biopsy showed infiltration of the esophageal mucosa by a malignant neoplasm consistent with mucosal melanoma. The tumor cells exhibited abundant cytoplasmic pigment, round-to-oval nuclei with prominent eosinophilic nucleoli, a high nuclear-to-cytoplasmic ratio and marked cellular atypia. Necrosis was observed surrounding the tumor nests. Immunohistochemistry results were as follows: Melan-A, positive; SOX-10, positive; S-100, focally positive; HMB-45, negative; cytokeratin, negative; p63, negative; CD117, negative; CEA, negative; Ki-67, positive (approximately 40%). The pathological diagnosis was primary esophageal melanoma. BRAF/MEK mutation analysis was negative. In this context, she was evaluated by Dermatology, ruling out cutaneous and ocular melanoma as the primary tumor. The positron emission tomography/CT (PET-CT) scan demonstrated increased uptake in the lesion located in the middle/distal third of the esophagus, highly suggestive of malignancy, with a maximum standardized uptake value (SUVmax) of 21.3 and no other significant hypermetabolic lesions visualized (Figure 4).

The patient's general condition progressively deteriorated, leading to complete dysphagia for solids and liquids one month after diagnosis. Urgent hospitalization was required due to malnutrition and worsening anemia (at this stage with iron deficiency), and parenteral nutrition was started. Considering the patient's advanced age and significant frailty (with a Clinical Frailty Scale score of 6), a multidisciplinary oncology team decided against surgical treatment due to the high morbidity associated with the procedure and poor potential for recovery. Her frailty was a key factor in the decision that surgical intervention would likely cause more harm than benefit, prompting the team to prioritize less invasive measures. A conservative palliative and minimally invasive strategy was adopted and a prosthetic esophageal stent was therefore proposed and placed, resulting in improved dysphagia and restoration of oral feeding.

Following clinical improvement, first-line systemic treatment with pembrolizumab every six weeks (intravenous) was initiated; only one dose was administered. Despite initial symptomatic relief after stent placement, her condition rapidly deteriorated, with a recurrence of complete dysphagia and severe malnutrition due to stent migration. As her nutritional status severely declined, she was rehospitalized in an emaciated state and was unable to tolerate further systemic treatment. The team revisited her treatment plan, ultimately shifting focus towards best supportive care and exclusive symptom relief strategy due to her declining condition. The patient ultimately died approximately two weeks after starting systemic treatment and six months after her diagnosis. The patient’s advanced age, decreased physiological reserve and frailty played a crucial role in her management, shaping treatment decisions that focused on balancing disease control with quality of life.

## Discussion

PMME is a rare type of esophageal cancer, originating from melanocytes, which are typically found in the skin [2,4,8]. It was officially recognized in 1963, when de la Pava et al. discovered the presence of melanocytes in the esophageal epithelium during autopsies [2]. Despite the identification of esophageal melanocytes in a small portion of the population, the pathogenesis and risk factors for developing PMME remain poorly understood, largely due to the unknown reason for the presence of melanocytes in the mucosa [4,9]. It is more common in men, with a male-to-female ratio of 2:1 [6,10,11], and most cases are diagnosed between the ages of 60 and 70 [6]. Unlike esophageal squamous cell carcinoma, there is no correlation with specific risk factors such as alcohol or tobacco consumption [12]. The case presented here is particularly noteworthy due to the female patient's age, exceeding the age range commonly reported in studies.

The most common symptom of PMME is progressive dysphagia, along with weight loss and nonspecific retrosternal pain, as seen in this case [5,6,10,11,13]. These symptoms, due to their nonspecific nature and association with a wide range of pathologies, can be easily overlooked, leading to delayed diagnoses [6,7,13]. The delay in the diagnosis of this case is notable, as the patient presented with symptoms that had been ongoing for approximately three months before further investigations were initiated.

In terms of its location, the middle and lower thirds of the esophagus are involved in about 90% of cases, likely due to the higher concentration of melanocytes in these regions, with invasion of the muscularis propria [5,6,10,12,14]. Tumors typically appear as a solitary mass that may be pigmented or non-pigmented (depending on the amount of melanin), with a lobulated polypoid form and the mucosa can remain intact or show occasional ulceration [2,6,11,15]. In our case, the lesion was located in the lower third of the esophagus and it presented with a violaceous/black color, in line with the usual macroscopic appearance of PMME. These are nonspecific findings and they highlight the importance of differential diagnoses, which should include conditions such as spindle cell carcinoma, leiomyosarcoma, esophageal lymphoma, and Kaposi's sarcoma [5,15]. However, the most common differential diagnosis is clear cell sarcoma [9]. The presence of ulceration is associated with a poor prognosis, although it was not observed macroscopically in this particular case [12]. Additionally, the presence of periesophageal lymph node metastasis does not correlate with tumor invasion [14].

Microscopically, the tumor cells display different sizes and shapes, including round, polygonal, or irregular forms, often containing melanin granules within their cytoplasm. These cells show a diffuse growth pattern and can infiltrate the submucosa and even extend into the muscularis propria [16].

The diagnosis of PMME is complex and requires a combination of clinical evaluation, auxiliary diagnostic tests, biopsy, and immunohistochemical studies. A multidisciplinary approach is essential for diagnosis and treatment [4,11,12,15]. Initially, a CT scan is useful for assessing tumor extent, but it does not characterize lesions specifically [5,12,13,15]. In this case, a chest X-ray was initially performed, revealing superior mediastinal widening and tracheal deviation, which led to a subsequent CT scan. To facilitate a thorough assessment and macroscopic observation of the lesion, an upper endoscopy is crucial. This also allows for biopsies, which are essential for diagnosis [5,12,15].

Immunohistochemistry is crucial for the definitive diagnosis of melanoma, with markers such as Melan-A, SOX-10, HMB-45, and S-100 used to identify the tumor type [2,11,13]. The S-100 protein was initially used for melanoma diagnosis, but HMB-45, which indicates active melanosome formation, has been proven more specific [17]. Melan-A is another marker found to be positive in a small percentage of HMB-45-negative melanomas, as it was detected in our case. Cytokeratin is generally negative, with positivity reported in only 7% of melanoma cases [17,18]. While S-100 is the most sensitive marker for melanoma (sensitivity 97%-100%, specificity 75%-87%), HMB-45 and Melan-A provide good specificity (HMB-45 sensitivity 69%-93%, Melan-A sensitivity 95%-100% and specificity 75%-92%), though their sensitivity is lower [17,18]. As for SOX-10, it is a transcription factor that regulates the differentiation and development of melanocytes and is also considered to be essential for their specification, maturation and maintenance [18]; therefore, when positive, it reflects the tumor's melanocyte lineage and supports the diagnosis of melanoma. Combining these markers in a larger immunohistochemical panel, along with additional markers for differential diagnosis (e.g., CEA, cytokeratin, CD117, p63), can significantly improve diagnostic accuracy. Furthermore, in tumors lacking macroscopic characteristics, the presence of these markers (Melan A, SOX-10, HMB-45 and S-100) is the key diagnostic method, especially in amelanotic tumors [2]. Amelanotic tumors are challenging to diagnose as they lack melanin, but immunostaining can still reveal melanoma markers [11]. In our case, the positive results for Melan-A and SOX-10, along with focal positivity for S-100, strongly suggested PMME. Negative results for epithelial markers (cytokeratin, p63, CEA) and CD117 helped exclude differential diagnoses such as carcinoma or gastrointestinal stromal tumor. Additionally, the Ki-67 index of 40%, reflecting moderate proliferation, correlates with the aggressive clinical course and rapid deterioration observed in our patient.

Finally, PET-CT is helpful in detecting metastases, with its sensitivity being directly proportional to the tumor size [5,12].

The case presented highlights the challenges of diagnosing esophageal malignant melanoma and the importance of early and multidisciplinary management. After a strong suspicion of PMME, it is important to rule out the involvement of other organs as the primary cause [13]. In this case, a comprehensive and multidisciplinary assessment was performed to rule out cutaneous or ocular melanoma as the primary cause.

Currently, there is no standardized classification (tumor-node-metastasis, or TNM, staging system) for PMME due to the rarity of diagnosis [9,10]. The disease can spread via hematogenous and lymphatic routes, with the liver being the most common site of metastasis, followed by mediastinum, mediastinal lymph nodes, lung, and brain [6].

Surgical resection consisting of esophagectomy with three-field lymph node dissection (periesophageal, mediastinal and celiac trunk) is the most common treatment, although it is only an option in non-metastatic situations [7,10,11,14]. However, the risk of recurrence is extremely high after the initial staging surgery, confirming the aggressive nature of PMME and the importance of adjuvant therapy [10,13]. Treatment decision could be difficult given the fact that esophagectomy is a highly aggressive procedure associated high morbidity [11]. Our patient was too frail to undergo primary surgical intervention, despite having a localized tumor. The role of systemic therapy for metastatic or unresectable PMME is still uncertain [10,14]. However, the current recommendation for systemic treatment is immunotherapy drugs, such as nivolumab, ipilimumab, and pembrolizumab [2,14], which appear to be promising options in patients with advanced PMME [14]. Treatment options for metastatic melanoma have improved significantly in the past five years with the development of targeted therapies and immunotherapies [10], but studies demonstrate a lower response rate to immunotherapy when compared to cutaneous melanoma [11]. Due to the availability of targeted therapies, BRAF mutations should be investigated, although they occur less frequently in mucosal than in cutaneous melanomas [4,11].

The prognosis for esophageal melanoma is unfavorable, with most patients dying within a few months [4,9,11]. Despite the poor prognosis, complete surgical resection with negative pathological margins is associated with a better outcome, but there is no evidence that radical resection improves survival [4]. In agreement with the view expressed in many case reports, studies emphasize the importance of early detection in managing PMME, though its effect on long-term survival remains uncertain. Cheng et al.*, *after the retrospective staging of patients according to the 2002 American Joint Committee on Cancer (AJCC) TNM classification, reported that stage II patients who underwent surgery had a one-year overall survival rate of 100% and a two-year rate of 75%. However, patients with stage III-IV disease had a much poorer prognosis, with a one-year overall survival of 0% [19]. Similarly, Harada et al. found better outcomes in patients without lymph node involvement, with a median survival of 34.5 months. Those without lymph node metastasis had a significantly longer disease-free survival, while patients with metastasis relapsed within a year (hazard ratio = 13.3, P = 0.009) [20]. These findings collectively suggest that early diagnosis, particularly before lymph node involvement, may improve short-term outcomes and enhance survival rates. However, the aggressive progression and high metastatic potential of PMME indicate that early detection alone may not significantly alter long-term prognosis. Therefore, while early surgical intervention, particularly when combined with neoadjuvant immunotherapy [10], may offer benefits in managing PMME, further large-scale studies are needed to develop comprehensive treatment strategies that can effectively improve survival outcomes.

## Conclusions

PMME is a rare and aggressive disease that presents significant diagnostic challenges and rapid clinical deterioration, especially in elderly patients. Early detection of the disease and a multidisciplinary approach are crucial for ensuring the short-term outcome for the patient. Although surgery is the only curative option, the challenging anatomical location of the tumor makes surgery a high-risk procedure. Systemic treatment for PMME remains uncertain, and while immunotherapy has shown success in cutaneous melanoma, its efficacy in mucosal melanoma is still unproven.

In this case, the patient’s advanced age and frailty were central to the treatment decisions, as the risks of aggressive therapies outweighed the potential benefits. The shift towards palliative care reflected the need to prioritize quality of life in such patients, underscoring the importance of individualized treatment. It is essential to note that our current understanding of this disease is primarily based on case studies and observations, with limited evidence from clinical trials. Given the rarity of PMME, conducting large cohort studies remains a significant challenge. However, alternative research approaches such as multicenter case registries and collaborative retrospective and prospective studies across hospitals, cancer centers, and academic institutions could help provide valuable insights into the disease. Additionally, forming an international consortium dedicated to rare esophageal cancers, including PMME, would foster cross-border collaborations and accelerate research into potential biomarkers, genetic profiles, and novel therapeutic approaches. By adopting these collaborative and innovative approaches, the medical community can advance the understanding of PMME and improve patient outcomes, despite the challenges posed by its rarity.
